# Hemocompatibility of Carbosilane Dendrimers as a Therapeutic siRNA Delivery System across Blood–Brain Barrier

**DOI:** 10.1021/acsami.5c12952

**Published:** 2026-02-10

**Authors:** Serafin Zawadzki, Simon Suty, Elżbieta Okła, Paula Ortega López, F. Javier de la Mata, Iveta Waczulikova, Maksim Ionov, Maria Bryszewska, Katarzyna Miłowska

**Affiliations:** † Department of General Biophysics, Faculty of Biology and Environmental Protection, 49602 University of Lodz, 141/143 Pomorska St., Lodz 90-236, Poland; ‡ Bio-Med-Chem Doctoral School of the University of Lodz and Lodz Institutes of the Polish Academy of Sciences, University of Lodz, Matejki 21/23, Lodz 90-237, Poland; § Department of Nuclear Physics and Biophysics, Faculty of Mathematics, Physics and Informatics, 37864 Comenius University, Mlynska Dolina F1, Bratislava 842 48, Slovakia; ∥ Department of Organic and Inorganic Chemistry, IQAR, University of Alcalá, Madrid 28805, Spain; ⊥ Networking Research Center on Bioengineering, Biomaterials and Nanomedicine (CIBER-BBN), Madrid 28029, Spain; # Ramón y Cajal Health Research Institute (IRYCIS), Madrid 28034, Spain; ∇ Faculty of Medicine, Collegium Medicum, Mazovian Academy in Plock, 2 Dabrowskiego Sq, Plock 09-402, Poland

**Keywords:** carbosilane dendrimer, nanoparticles, siRNA, drug delivery system, hemocompatibility, immunocompatibility

## Abstract

The development of nanocarriers offers a promising strategy for the delivery of therapeutics to the central nervous system. However, the clinical translation of nanosystems hinges on their interactions with blood components, which not only dictate their biodistribution and therapeutic efficacy but also may pose potential risks to hemostasis. In this study, we assess the hemocompatibility of a novel, third-generation PEGylated carbosilane dendrimer (G3Si PEG6000) and its dendriplex designed for siRNA delivery across the blood-brain barrier pertinent to Alzheimer’s disease. Utilizing a comprehensive array of advanced analytical techniques, we assess cellular responses, cytokine expression, hemorheological properties, hematological parameters, and coagulation dynamics within a physiologically relevant environment. Our findings demonstrate that the investigated nanosystem elicits changes in blood rheology, immune recognition, and the intrinsic coagulation cascade, yet these effects remain below thresholds associated with clinically significant adverse outcomes. Hemolysis was ∼8-fold lower for dendriplexes than the dendrimer in PBS at the highest concentration (accordingly 3.5 ± 0.14% vs 27.46 ± 4.66%, 24 h), in 55% plasma, both formulations were nonhemolytic across all concentrations. Whole blood viscosity increased by up to ∼11% (dendrimer) and ∼16% (dendriplex) relative to the control. At 10 μM, the dendrimer approximately doubled the aPTT, whereas the corresponding dendriplex increased the aPTT by ∼30% of the control. Importantly, neither adverse effects on red blood cell and platelet indices nor toxicological responses in white blood cells were observed under the tested conditions. These findings not only support the translational potential of the studied nanosystem for therapy but also emphasize the critical role of the therapeutic cargo and the formation of a biomolecular corona in shaping the nanocarrier’s biological identity and its subsequent interactions within the bloodstream. The results provide a compelling scientific basis for advancing this platform in further investigations.

## Graphical abstract

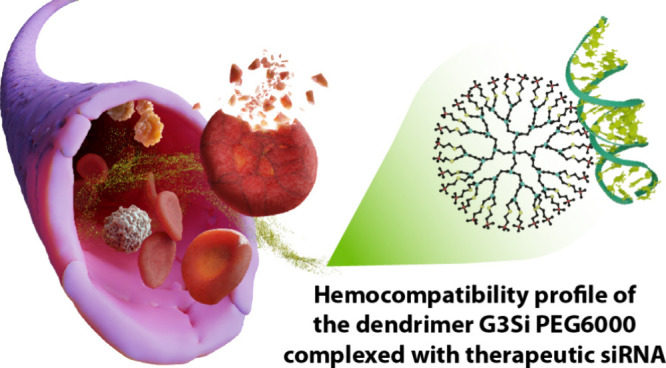

## Introduction

The blood–brain barrier (BBB) presents a significant challenge to the treatment of neurological disorders. This is due to the restrictive nature of the BBB, which impedes the delivery of therapeutics to the central nervous system (CNS). Nanotechnology offers a promising solution by employing nanovectors to cross the BBB and deliver therapeutic agents to the target site. The success of nanocarriers is heavily dependent on their biocompatibility. Hemocompatibility is particularly crucial given that many therapeutic strategies involve parenteral administration, which bypasses the gastrointestinal tract and often results in direct exposure of the bloodstream to administered substances. This approach offers the advantage of systemic delivery, circumventing first-pass metabolism and potentially enhancing therapeutic effects. However, the blood environment is highly complex, and the interactions of nanoparticles with blood components can significantly influence the fate and efficacy of the nanovector particles (further nanovectors) as well as constitute risks to hematological balance. Therefore, blood-contacting nanomaterials must be tested for adverse interactions with the blood components. In order to assess the hemocompatibility of nanoparticles, it is imperative to recognize their possible impact on the properties and functions of individual blood components within a highly interactive environment where all elements are in constant communication. Figure 1 illustrates nanovectors crossing the BBB, emphasizing their interactions with blood components.

**Figure 1:**
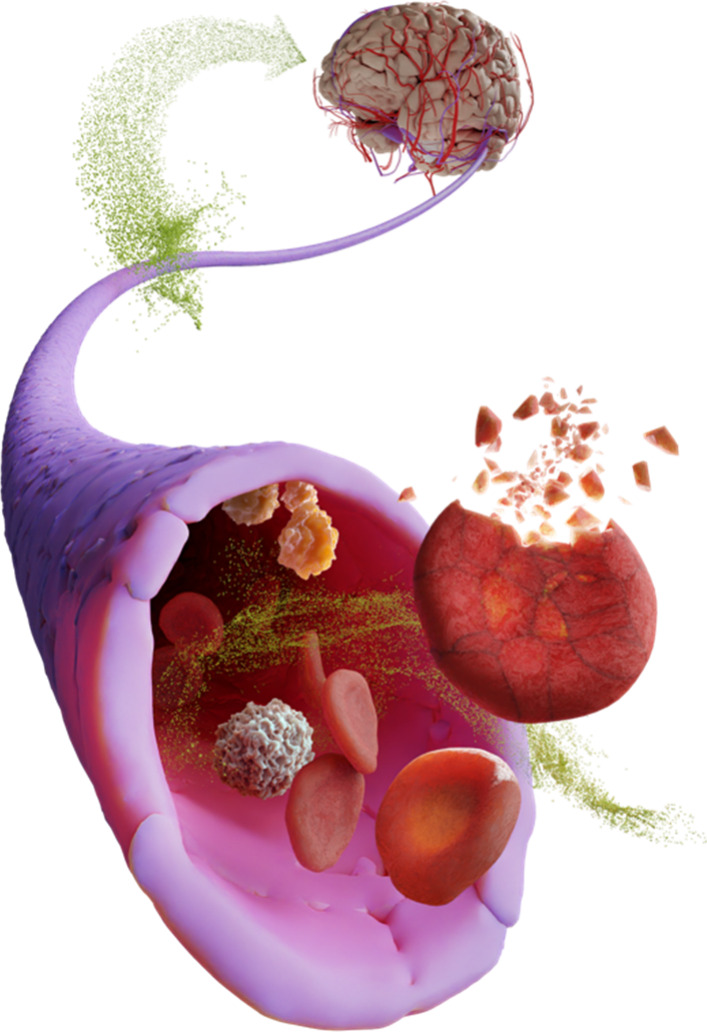
Illustration depicts nanovectors (in green) after intravenous injection, interacting with blood components such as red blood cells, blood platelets, and immune cells potentially, leading to adverse effects like hemolysis, clot formation, and immune response.

Erythrocytes, the most common blood cells, perform vital functions beyond oxygen and carbon dioxide transport, including roles in hemostasis, thrombosis, and the regulation of blood viscosity. Their distinctive rheological properties contribute to the non-Newtonian behavior of blood, influencing its flow dynamics and overall circulatory function.^1,2^ The interactions of nanoparticles with erythrocytes can disrupt the delicate physiological balance, leading to a cascade of intertwined and mutually reinforcing adverse events, including hemolysis, erythrocyte deformation, aggregation, and an increased risk of thrombosis. Nanoparticle-induced hemolysis, while reducing erythrocyte numbers and potentially leading to life-threatening anemia, also causes the release of hemoglobin, which can contribute to endothelial dysfunction and thrombus formation through nitric oxide sequestration.^3–6^ Nanoparticle-induced hemotoxicity can significantly alter the blood rheology. Hemolysis can often lead to a decrease in blood viscosity, which in turn increases the risk of bleeding, especially in patients with fever. Conversely, nanoparticle-mediated erythrocyte deformation, often leading to an increased RBC membrane rigidity, can elevate blood viscosity. This increased viscosity contributes to a range of hematological and neurological disorders by promoting microvessel occlusion, altering blood flow patterns, and facilitating platelet activation, ultimately increasing thrombotic potential.^7,8^ Furthermore, nanoparticles can induce phosphatidylserine exposure on erythrocyte membranes, creating a procoagulant surface that promotes platelet adhesion and increases the risk of thrombosis, thereby influencing coagulation.^9–14^

Blood platelets are the second most abundant cell type in the blood. Formerly regarded solely as regulators of hemostasis, platelets are now vastly acknowledged for their involvement in various crucial functions, including vascular inflammation, combating infections, innate immunity, regulating tumor growth, and promoting angiogenesis.^15,16^ This expanded understanding necessitates careful consideration of platelet interactions with nanovectors, as their encounters can trigger unintended activation of signaling pathways, potentially leading to consequences beyond blood clot formation. Notably, the negatively charged sialic acid residues present on the platelet surface exhibit a strong affinity for cationic nanoparticles, which can induce platelet aggregation via cross-bridge formation.^17^ A critical consequence of nanoparticle-induced platelet activation is the release of pro-inflammatory mediators, including β-thromboglobulin, and the translocation of the membrane protein P-selectin, subsequently triggering leukocyte recruitment, which can further amplify the inflammatory response.^18–20^

Leukocytes are essential components of the immune system that orchestrate complex recognition and response mechanisms, leading to the neutralization or elimination of foreign entities. Depending on their biological and synthetic identity, nanoparticles may be perceived as foreign, self-conscious, or evade immune recognition entirely. If recognized as foreign, nanoparticles can trigger a defensive immune reaction, leading to their engulfment by macrophages, neutrophils, dendritic cells, or monocytes.^21^ The mononuclear phagocytic system (MPS) and reticuloendothelial system (RES) contribute to the accumulation of nanoparticles in off-target organs such as the liver, spleen, and lungs.^22^ Due to their role in clearing foreign particles from the bloodstream, which often results in the high accumulation of nanoparticles inside immune cells, leukocytes are particularly susceptible to nanoparticle toxicity. Nanoparticles can interfere with chemotaxis, cytokine secretion, oxidative bursts, and phagocytosis. Additionally, they may induce more severe adverse effects, such as morphological changes, DNA damage, and apoptosis, leading to immune system pathologies.^23^

Given the critical role of hemocompatibility in the successful application of nanocarriers, it is essential to thoroughly evaluate their effect on blood physiology. By providing a holistic understanding of their interactions with blood components, we can identify potential adverse effects and optimize their design for improved biocompatibility and therapeutic efficacy. In our previous work, we have synthesized, characterized, and chosen a single dendrimer best fit for delivering therapeutic agents across the blood-brain barrier.^24^ However, for every nanovector, its potential for clinical translation is contingent on its hemocompatibility profile in the range of effective doses. The current broad-based study aims to assess the hemocompatibility of the third-generation, positively charged, PEGylated carbosilane dendrimer and its complex with siRNA targeting the apolipoprotein E 4 (APOE4) gene.^25^ This gene-targeting nanosystem is a potential therapeutic target for Alzheimer’s disease, and understanding the hemocompatibility of this system is crucial for its development as a therapeutic approach. The findings of our analysis encompassed valuable insights, including erythrocyte interactions, immune cell responses, hemorheology, and multiple hematological and coagulation parameters. These effects were examined across three distinct experimental conditions: in the absence of plasma, with plasma present, and with whole blood. This approach allowed us to assess how plasma and blood components influence the studied nanovector system’s interactions with blood morphotic elements, providing a comprehensive view of its biological activity. To contextualize the present hemocompatibility analyses, we captured G3Si PEG6000 nanosystem with biomolecular corona binding signatures and morphology under conditions approximating the intended plasma milieu.^26^ The findings contribute significantly to a more profound understanding of the safety and efficacy of the nanocarriers studied for potential clinical applications and guide future research efforts toward more in-depth investigation of specific areas of concern.

## Materials and Methods

### Dendrimer

Dendrimer with a silicon atom core, surface tertiary ammonium groups, and single polyethylene glycol (PEG) (6000)-G3Si PEG6000 with chemical formula C_601_H_1324_I_30_N_31_O_135_S_32_Si_29_, and a molar weight of 16,794.81 g/mol is presented in Figure 2. The studied dendrimer was diluted in a phosphate buffer solution of 7.4 pH. The synthesis and biophysical characterization of the dendrimer, along with its complex with siRNA, were described in previous work.^24^

**Figure 2:**
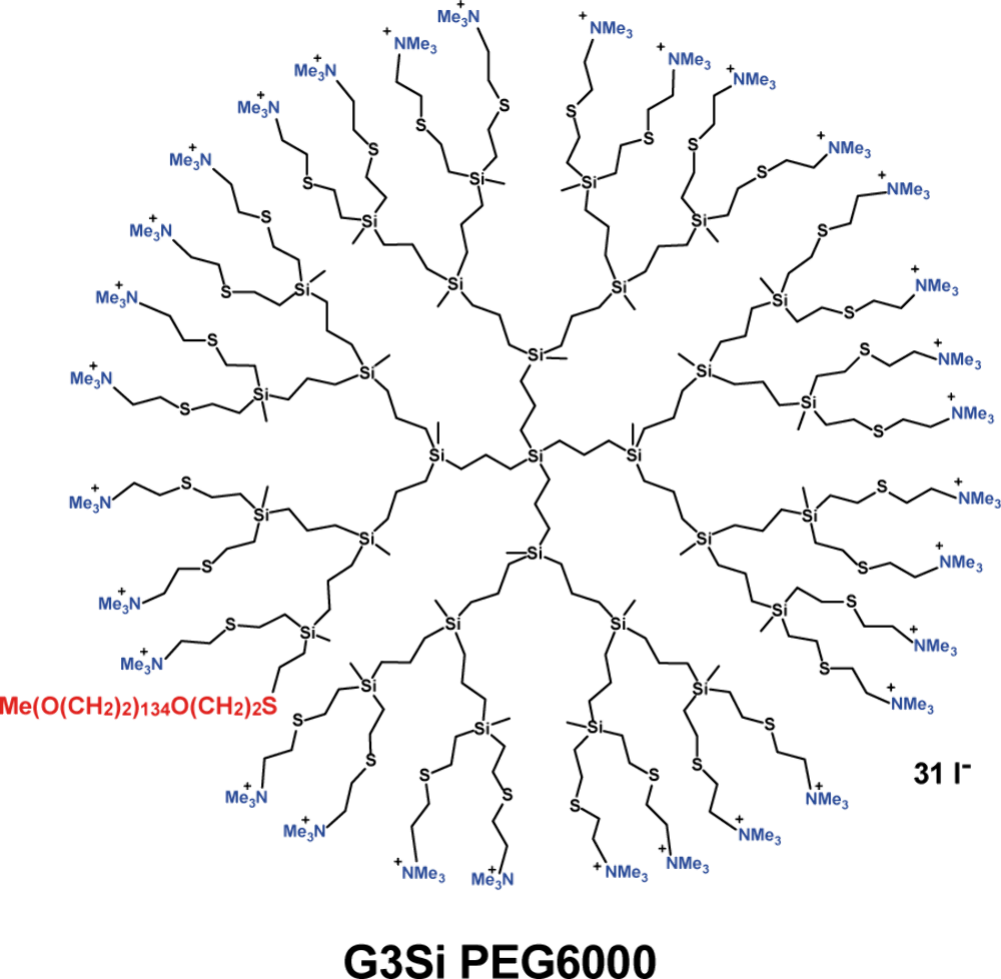
Structure of the studied dendrimer G3Si PEG6000.

### siRNA

The following siRNA was used in the present study (Dharmacon Inc., Lafayette, CO, USA): Sense: 5′-GAUUACCUGCGCUGGGUGCUU; Antisense: 5′-PGCACCCAGCGCAGGUAAUCUU.

### Other Reagents

Fetal bovine serum (FBS), RPMI-1640 medium, and resazurin were obtained from Biowest, Nuaillé, France. Sigma-Aldrich (Saint Louis, MO, USA) supplied Histopaque-1077, MILLIPLEX Human Cytokine Panel, phosphate-buffered saline (PBS) tablets, DPH (1,6-diphenyl-1,3,5-hexatriene), TMA-DPH (N,N,N-trimethyl-4-(6-phenyl-1,3,5-hexa-triene-1-yl), and 1% penicillin-streptomycin. Other chemical reagents, including sodium dihydrogen phosphate and disodium hydrogen phosphate, were obtained from Chempur (Piekary Śląskie, Poland). These chemicals were of analytical grade, and solutions were prepared using water purified with the Mili-Q system.

### Dendriplex Formation

Our prior research provides insights into the dendrimer/siRNA complexation process. We characterized Dendrimer G3Si PEG6000 and determined its optimal dendriplex saturation at a molar ratio of dendrimer to siRNA of 2.5:1.^24^ In this research, we chose dendrimer G3Si PEG6000 concentrations ranging from 0.1 to 10 μM and 0.04 to 4 μM for the siRNA in dendriplex. The dendrimer/siRNA complexes were incubated in PBS at room temperature for 20 min to ensure the formation of complexes.

### Hemolysis

Blood donations from healthy donors were collected by the Regional Centre for Blood Donation and Hemotherapy in Lodz into CPD (citrate–phosphate–dextrose) anticoagulant blood-bag systems. All experiments used individual-donor plasma (no pooling was applied). Erythrocytes were separated from blood plasma and white blood cells (WBCs) and washed three times with PBS by centrifugation at 3000 rpm, 10 min, at 4 °C. Erythrocytes were used immediately after isolation. To evaluate the effects of G3Si PEG6000 and its dendriplexes on erythrocytes, the studied compounds were added to red blood cells (RBCs, 2% hematocrit) and incubated at 37 °C for either 3 or 24 h, followed by centrifugation at 3000 rpm for 10 min. Hemolysis was assessed by measuring the hemoglobin content in the supernatant at a wavelength of 540 nm by using a multiwell plate reader (BioTek PowerWave HT, BioTek Instruments, Inc., Winooski, VT, USA). The percentage of hemolysis was calculated using eq 1:

$$\%\;\mathrm{Hemolysis}=\frac{A_{s}}{A_{c}}\times 100\%$$ 1

where *A*_s_ is the absorbance of cells treated with dendrimer or dendriplex, and *A*_c_ is the absorbance of erythrocytes in water (100% of hemolysis). The experiment was performed in 3 repetitions.

### Hemolysis Assay in the Presence of Plasma Proteins

Human plasma was separated from blood by centrifuging at 4200 rpm for 15 min. All experiments used individual-donor plasma (no pooling was applied). The dendrimer and dendriplex were preincubated in a 55% plasma solution (v/v) in PBS at 37 °C and time of 1 h as it is often considered sufficient for corona formation equilibrium.^27^ Following this preincubation, the samples were mixed with isolated RBC and plasma to create 2% hematocrit and 55% plasma solution (v/v) in PBS and were further incubated for 3 or 24 h. The same procedure was used to assess hemolysis in the presence of plasma, as described earlier. The experiment was performed in 3 repetitions.

### PBMC Cytotoxicity

PBMCs were isolated using Histopaque from the buffy coat of 3 healthy blood donors obtained from the Regional Centre for Blood Donation and Hemotherapy in Lodz into CPD (citrate–phosphate–diastereose) anticoagulant blood-bag systems. All experiments used individual-donor plasma (no pooling was applied). After isolation, the cells were resuspended in RPMI-1640 medium supplemented with 10% FBS (heat-inactivated), 1% penicillin, and streptomycin. The cells were seeded on 96-well black plates in a density of 1 × 10^4^ cells/well (37 °C, 5% CO_2_). To evaluate the effects of G3Si PEG6000 and its dendriplex on PBMC, they were incubated for 24 and 48 h. After incubation, a resazurin solution in PBS was added for 2 h, obtaining a final concentration of 0.125 mg/mL per well. Fluorescence measurements were read at wavelengths of λ_ex_ = 530 nm and λ_em_ = 590 nm using a multiwell plate reader (BioTek PowerWave HT, BioTek Instruments, Inc., Winooski, VT, USA). The cell viability percentage was calculated using eq 2:

$$\%\;\mathrm{Viability}=\frac{A_{s}}{A_{c}}\times 100\%$$ 2

where *A*_s_ is the absorbance of cells treated with dendrimer or dendriplex, and *A*_c_ is the absorbance of control cells (untreated). The experiment was performed in 4 repetitions.

### PBMC Cytokine and Chemokine Expression Pattern

To evaluate secretion of cytokines and chemokines by PBMCs in response to dendrimer G3Si PEG6000 and its dendriplex, PBMCs were isolated, seeded as described earlier, and incubated with the tested compounds at 37 °C and 5% CO_2_. After 48 h incubation, cell culture supernatants were collected and analyzed using the Luminex MAGPIX analyzer (Luminex, Austin, Texas, United States) to assess IFNα 2, IL-6, IL-8, IL-10, IL-12p40, and TNFα levels. All procedures were strictly followed according to the manufacturer’s protocols. The studies were carried out at the Research Laboratory CoreLab of the Medical University of Lodz. The experiment was performed in 7 repetitions.

### PBMC Cytokine Expression in the Presence of Plasma Proteins

Human plasma was separated from blood by centrifuging it at 4200 rpm for 15 min. All experiments used individual-donor plasma (no pooling was applied). Dendriplexes and the not complexed dendrimer were preincubated in a 55% plasma solution (v/v) in PBS for 1 h at 37 °C. Following this preincubation, the samples were mixed with isolated PBMCs and 55% plasma solution (v/v) in RPMI-1640 and were further incubated for 48 h. The same procedure was used to assess PBMC cytokine expression in the presence of plasma, as described earlier. The experiment was performed in 6 repetitions.

### Hemorheology

Blood samples were collected from three healthy male volunteers (aged 20–30 years) with no known severe health conditions following an overnight fast. Males were selected for this study due to their physiologically higher baseline hematocrit than females, making them more susceptible to the adverse effects of elevated blood viscosity. This study was conducted in accordance with the ethical principles set forth in the Declaration of Helsinki, and the study protocol was approved by the Institutional Ethics Committee at the St. Elizabeth Cancer Institute in Bratislava (approval no. 03-2020/EK ÓSA, signed on March 4, 2020). To maintain physiological conditions, blood samples were immediately placed in a water bath maintained at a constant temperature of 37 °C using a thermostatic controller (Polystat Control CC1, Huber, Berching, Germany). Gentle manual agitation of the tubes every 10 min prevented sedimentation. Rheological properties were subsequently analyzed by using a modular compact rheometer (MCR 102, Anton Paar, Graz, Austria) equipped with a double-gap measuring system (DG 26.7/Ti). All measurements were conducted at 37 °C, maintained by the rheometer’s integrated Peltier temperature control system. Viscosity measurements were performed over a shear rate range of 1–1000 s^–1^, with the duration of each measurement point determined automatically by the instrument to ensure the acquisition of stable viscosity values.

### Erythrocyte Membrane Fluidity

To evaluate the impact of G3Si PEG6000 and its dendriplex on erythrocyte membrane fluidity and their ability to interact with both hydrophobic and hydrophilic regions of the erythrocyte lipid bilayer, fluorescence anisotropy measurements were performed using two probes: DPH (1,6-diphenylhexatriene) and its hydrophilic derivative TMA-DPH (1-(4-Trimethylammoniophenyl)-6-phenyl-1,3,5-hexatriene p-toluenesulfonate). The experiments were conducted utilizing 0.05% erythrocyte solution in PBS at 37 °C in 1 cm path-length quartz cuvettes using a PerkinElmer LS-55 spectrofluorometer (PerkinElmer, Waltham, MA, USA). The excitation and emission wavelengths were set to 348 and 358 nm for DPH and 426 and 428 nm for TMA-DPH, respectively. The experiment was performed in 6 repetitions.

### Hematological and Coagulation Blood Parameters

Blood was obtained from 3 volunteers as described in the rheology section. The samples were collected in two separate tubes: with K2EDTA for hematological analysis and with sodium citrate for coagulation analysis. For hematological analysis, blood samples in K2EDTA tubes were incubated at room temperature, protected from light, for 3 h before analysis using a COULTER DxH 800 automatic hematology analyzer (Beckman Coulter Inc., Brea, CA, USA). Coagulation parameters were immediately assessed using an ACL Top 500 CTS automatic hemostasis analyzer (Instrumentation Laboratory, Bedford, MA, USA). This methodology enabled the simultaneous quantitative assessment of hematological parameters using a combination of multiple techniques. Impedance analysis, based on Coulter’s principle, measured and sized cells by detecting changes in electrical resistance as they traversed a small aperture. Radiofrequency analysis provided insights into the internal conductivity of cells, offering information about cellular density and granularity through the application of a high-frequency electromagnetic field. Flow cytometric light scatter was employed to detect light scattered by cells at various angles, yielding data on the cell size (forward scatter) and internal complexity or granularity (side scatter). Additionally, spectrophotometry was used to determine hemoglobin levels by measuring the absorbance of specific wavelengths of light, while supravital staining allowed the examination of living cells stained with dyes that penetrated their membranes without causing cell death.

### Statistical Analyses

Statistical analyses and data visualization were performed using Prism version 10.4.2 (GraphPad Software, Boston, MA, USA). The Shapiro–Wilk test was used to assess the normality of data distributions. Depending on experimental design, either one-way or multiple-way ANOVA was applied to assess differences in group means. Where ANOVA indicated statistically significant main effects, appropriate post hoc multiple comparison tests were selected based on the structure of the comparisons: Tukey’s test was used for evaluating all pairwise group differences, Dunnett’s test for comparisons of multiple treatments against a single control group, and Šidák’s test for a limited set of planned comparisons. In case of non-normally distributed data or ordinal outcomes the simple effects were assessed using nonparametric Kruskal–Wallis test followed by Dunn’s multiple comparisons test. All tests were two-tailed and performed at a significance level α = 0.05. In our graphical representations, significance is denoted by asterisks: one asterisk (*) indicates a *p*-value < 0.05, two asterisks (**) represent a *p*-value < 0.01, and three asterisks (***) denote a *p*-value < 0.001.

## Results and Discussion

The earliest experiments leading to recognition of the importance of hemocompatibility had their rudimentary beginnings in the 19th century. Hewson observed that stationary blood exhibited different clotting behavior within a living vein segment compared to an open bowl.^28^ Later, Virchow showed that in addition to the blood-material interface, other factors like blood composition and flow influence measurable blood clotting.^29^ The foundation for modern testing methods was established in the 1970s and 80s through a series of seminars and consensus conferences.^30,31^ This groundwork led regulatory bodies like the USA Food and Drug Administration (FDA) and the European Medicines Agency (EMA) to establish official guidelines. These guidelines recommend specific assays that manufacturers must perform to ensure the safety of blood-exposed foreign materials. Further solidifying these practices, the ISO 10993-4 standard (issued in 2002 and updated in 2009 by the International Organization for Standardization, the American National Standards Institute, and the Association for the Advancement of Medical Instrumentation) provides a guideline for hemocompatibility testing.^32^ Standardized in vitro tests, while invaluable for ensuring comparability and regulatory alignment, often fail to address nanoparticles’ nuanced behaviors. To bridge this gap, tailored approaches that thoroughly investigate nanoparticle-specific properties and their interactions with biological systems must complement existing standards, ensuring that results remain comparable and facilitate regulatory evaluation while capturing the complexities of nanoparticle behavior. Such a balanced strategy enables the reliable assessment of safety and efficacy, paving the way for successfully integrating nanoparticle-based medical products into clinical and commercial use.^33,34^

### Hemolysis

Hemolytic properties are typically the primary parameters assessed when evaluating the hemocompatibility of nanovectors. In this study, we assessed the hemolytic properties by spectroscopic measurement of hemoglobin released from erythrocytes after 3 and 24 h treatment with G3Si PEG6000 dendrimer and its dendriplex (Figure 3). The most pronounced and statistically significant hemolytic effect was observed for 2.5 and 10 μM concentrations of the noncomplexed dendrimer, with the effect increasing over the duration of incubation. After 24 h, the 10 μM dendrimer concentration induced hemolysis equivalent to 27.46%, indicating the loss of integrity of the erythrocyte membrane or rupture accompanied by a leakage of hemoglobin. The complexation of the nanovector with siRNA significantly reduced hemoglobin release, demonstrating markedly lower hemotoxicity. The complexation of counter-anions of siRNA effectively neutralizes a fraction of the accessible amine charge, and PEGylation provides additional spatial repulsion and establishes a steric/hydration barrier, fundamentally altering the physicochemical properties of the cationic nanosystem.^35–37^ This reduction in effective positive surface charge lessens electrostatic docking between the nanoparticle and anionic cell membranes, such as those of erythrocytes, thereby the interfacial charge masking limits membrane perturbation and mitigates disruption of bilayer integrity, nanoscopic poration with ensuing hemolysis.^38^ The highest tested dendriplex concentration exhibited 3.5% hemolysis after the 24 h incubation period. This reduction of hemotoxicity can be attributed to the lower positive charge of dendriplexes compared to noncomplexed dendrimers. Since the surface of RBCs is negatively charged, the diminished electrostatic attraction between dendriplexes and the cell membrane minimizes disruption and subsequent hemoglobin leakage.

**Figure 3:**
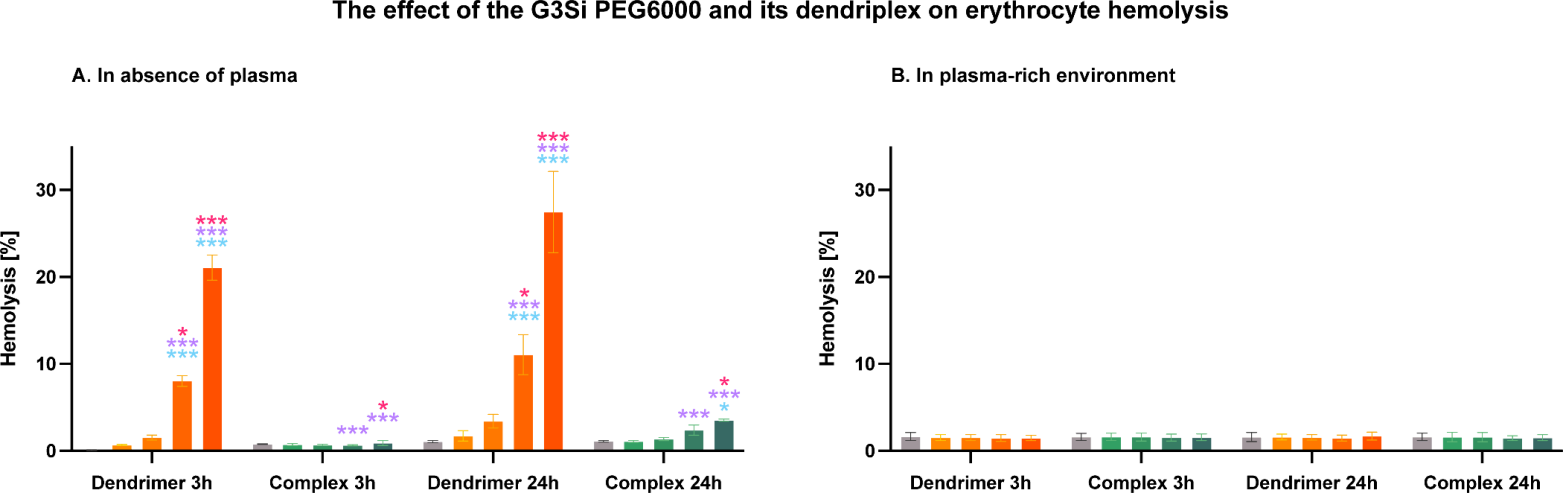
Hemolysis of erythrocytes treated with dendrimer G3Si PEG6000 and its dendriplex following 3 and 24 h incubation in PBS (A) and 55% plasma in PBS (B). A red asterisk denotes statistically significant effects attributed to incubation time; a violet asterisk indicates significant differences due to dendrimer-siRNA complexation; and a blue asterisk marks significant differences relative to the negative control.

While the assay provides valuable insights into cell type-specific responses, its current design does not reflect in vivo comparative toxicity endpoints. Many studies have explored the hemolytic properties of nanoparticles, but few have considered the influence of the biomolecular corona. The formation of a biomolecular corona on the nanoparticle surface is a critical factor that can mitigate hemolytic activity by masking the nanoparticle’s synthetic identity properties and altering its interactions with biological systems.^39,40^ Consistent with this view, our companion study on the same G3Si PEG6000 platform shows that the dendrimer engages albumin and transferrin through multivalent, entropy-favored binding with secondary/tertiary conformational modifications indicative of rapid corona establishment that passivates the interface and thus may attenuate direct RBC membrane disruption.^26^ To quantify the hemolysis suppression effect, the tested nanoformulations were preincubated with 55% plasma in PBS for 1 h prior to their addition to RBCs in a 55% plasma solution. This approach showed that while uncoated dendrimer G3Si PEG6000 had a significant hemolytic effect on RBC, the formation of a biomolecular corona interface on the particle surface protected the cells from being damaged. In PBS, G3Si PEG6000 induced 27.46 ± 4.66% hemolysis, while the dendriplex induced 3.50 ± 0.14%. In 55% plasma, reduced hemolysis for both formulations was reached at the level of the negative control (background), corresponding to relative reductions of ∼100%. These findings align with other studies investigating the effects of plasma protein layer on the nanoparticle surface on RBC hemolysis, which similarly reported reduced hemolytic activity.^41–44^ Upon plasma exposure, macromolecules rapidly assemble into a corona on cationic surfaces that masks cationic/hydrophobic motifs, lowers the effective surface potential, and establishes a hydrated steric layer. This interfacial remodeling shifts contacts from direct electrostatic engagement with the anionic, sialic-acid-rich RBC glycocalyx to protein-mediated interactions, thereby attenuating membrane perturbation, hemolysis, and eryptosis.^45–47^ Although some highly hydrophobic or strongly cationic nanoparticles can retain hemolytic activity after plasma exposure, the tested nanosystem, even in the siRNA-free form, did not exhibit such residual activity under 55% plasma conditions. This suggests that the presence of a biomolecular corona on the positively charged surface of the dendrimer G3Si PEG6000 can significantly diminish the hemolytic potential of these nanostructures. However, many nanovectors can still significantly impact erythrocyte function, even with limited direct hemolytic activity. These effects can manifest as erythrocyte aggregation or morphological deformation, both of which can disrupt hemorheology and impair blood flow, potentially leading to vascular complications.^23^

### Hemorheology

Hemorheology examines the influence of blood flow dynamics on hemostasis, primarily with particular attention paid to the interplay between shear rates and blood viscosity. Erythrocytes, due to their unique properties, are the primary contributors to blood viscosity and the non-Newtonian behavior of blood.^48^ Under low shear rates, the slow movement of blood, macromolecules dispersed in the plasma (such as fibrinogen), and the discoid shape of erythrocytes collectively promote erythrocytes to aggregate into stacked face-to-face “rouleaux” structures. As the diameter of vessels decreases and the shear rate increases, RBC aggregates gradually separate, causing a substantial viscosity drop of ∼10 s^–1^. Furthermore, a subsequent 4-fold viscosity decrease occurs due to a morphological transition in RBC.^49,50^ Erythrocyte physiological reversible aggregation plays a significant role in influencing the distribution of cellular components within the bloodstream. Moving mostly near the blood vessel center, erythrocytes force platelets and other particles toward the periphery. This process, known as axial margination, depends on blood viscosity and erythrocyte aggregation and influences the distribution of platelets, leukocytes, and other elements like nanovectors within the vasculature.^51–53^ All events taking place in flowing blood generate shear forces that influence blood behavior. Typical shear rates in vivo are approximately 20–200 s^–1^ for veins, 300–800 s^–1^ for large arteries, 500–1600 s^–1^ for arterioles, and 800–10,000 s^–1^ for atherosclerotic stenosed arteries.^54^ Increasing concentrations of the dendrimer and its dendriplex led to a notable increase in blood viscosity, particularly at lower shear rates. This effect is highly comparable between dendriplexes and noncomplexed dendrimers. In our in vitro study, at a shear rate of 1 s^–1^, the viscosity of a blood sample without the addition of tested compounds was equal to 16.48 ± 1.74 mPa s. After introducing the highest concentration of dendrimer G3Si PEG6000, the viscosity increased to 18.57 ± 2.17 mPa s, while with the addition of the highest concentration of dendriplex, it rose to 19.30 ± 1.56 mPa s. In higher shear rates, the effect is less visible. As shown in Figure 4, the viscosity of the samples decreases with increasing shear rates (decreasing vessel diameter), a phenomenon known as the Fåhræus–Lindqvist effect caused by the formation of a few-micron-thick cell-free layer adjacent to the vessel walls.^55,56^ Increased viscosity can reduce cardiac output and elevate peripheral resistance, leading to higher blood pressure and placing additional mechanical strain on the cardiovascular system. This can exacerbate conditions such as poor circulation, heart failure, and hematological and neurological disorders while significantly raising the risk of ischemic stroke.^51,52,57–59^ In our study, the increase in blood viscosity observed after the addition of the tested compounds within the concentration range does not reach levels likely to cause cardiovascular complications linked to hemorheology.^60^

**Figure 4:**
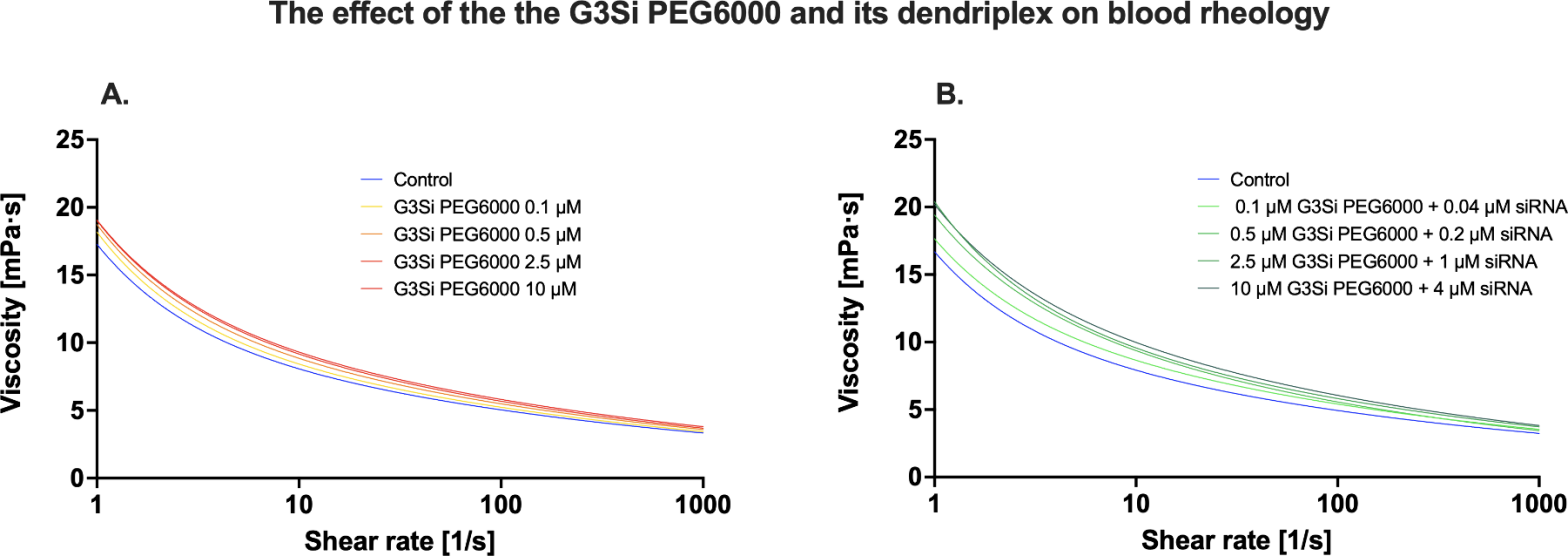
Changes of whole blood viscosity for different shear rate parameters following the addition of dendrimer G3Si PEG6000 (A) and its dendriplex (B). The Herschel-Bulkley model for non-Newtonian fluids was fit to the data.

Nanoparticles can have diverse effects on blood rheology depending on their size, shape, surface properties, and concentration. For example, amphiphilic phosphorene dendrons increase blood viscosity in a concentration-dependent manner, most likely mainly due to electrostatic interactions with cell membranes.^61^ Conversely, diesel exhaust particles, an environmental nanoparticle, can alter blood flow properties by damaging erythrocyte cell membranes, potentially reducing blood viscosity.^7^ This highlights the importance of considering nanoparticle-driven effects on hemorheology. As there are three main factors that can influence blood viscosity, namely, hematocrit, erythrocyte aggregation, and erythrocyte deformation, there are two most probable potential mechanisms that may lead to the increase of blood viscosity observed in our study. The tested dendrimer-based therapeutic system attached to the surface of erythrocytes may increase the value of the adhesion force acting between them or may cause the stiffening of the RBC membrane.

### Erythrocyte Membrane Fluidity

Nanovectors can significantly influence membrane fluidity by interacting with the RBC membranes’ lipid bilayer and structural proteins. These interactions may alter erythrocytes’ deformability and biconcave shape, which is critical for their role in maintaining proper blood viscosity and efficient microcirculation perfusion. Disruption of the double-lipid membrane or transmembrane protein organization by nanoparticles could hinder the integrity of membrane architecture, affecting their functions and lifespan and potentially leading to eryptosis.^62^ The influence of the G3Si PEG6000 dendrimer on erythrocyte membrane fluidity was assessed by fluorescence spectroscopy. The measurement of fluorescent anisotropy of TMA-DPH and DPH, respectively, reflects membrane fluidity of the hydrophilic surface and hydrophobic membrane core of the lipid bimolecular layer. Exposure to the G3Si PEG6000 dendrimer and its dendriplex resulted in decreased membrane rigidity (Figure 5), with a slightly greater effect observed in the hydrophilic regions compared to the hydrophobic core. This increased interaction with the hydrophilic membrane surface is likely due to the highly charged ionic nature of G3Si PEG6000 dendrimers, which exhibit hydrophilic properties that facilitate interactions with polar regions of the lipid bilayer.^63^ However, these same hydrophilic characteristics present a challenge for membrane penetration, as the dendrimers must overcome the substantial energy barrier imposed by the hydrophobic core of the lipid bilayer.^64^ This effect is more pronounced with the studied dendrimer than with its dendriplex, as the dendrimer alters membrane fluidity at a lower concentration compared to the dendriplex, underscoring the effect of dendrimer-siRNA interactions in modulating membrane fluidity. This observation aligns with hemotoxicity studies, which similarly highlight how the complexation of the dendrimer with siRNA modulates its interaction with biological membranes, reducing its impact on cellular integrity. While the lipid bilayer is highly adaptable and expandable, its ability to stretch is inherently limited, failing under tension beyond just a few percent deformation, highlighting its sensitivity to such interactions.^64^ The interaction of the studied dendrimer-based therapeutic system with isolated RBCs results in an increase in membrane fluidity rather than rigidity. Based on this observation, we conclude that the observed increase in blood viscosity after the addition of the dendrimer and its dendriplex, as seen in the hemorheological study conducted on whole blood, is more likely attributed to an increase in the adhesion forces between the RBC. A decrease in deformability, by contrast, would typically lead to a viscosity increase; however, this study indicates an increase in erythrocyte deformability, supporting the hypothesis of enhanced RBC adhesion as the primary mechanism.^65^

**Figure 5:**
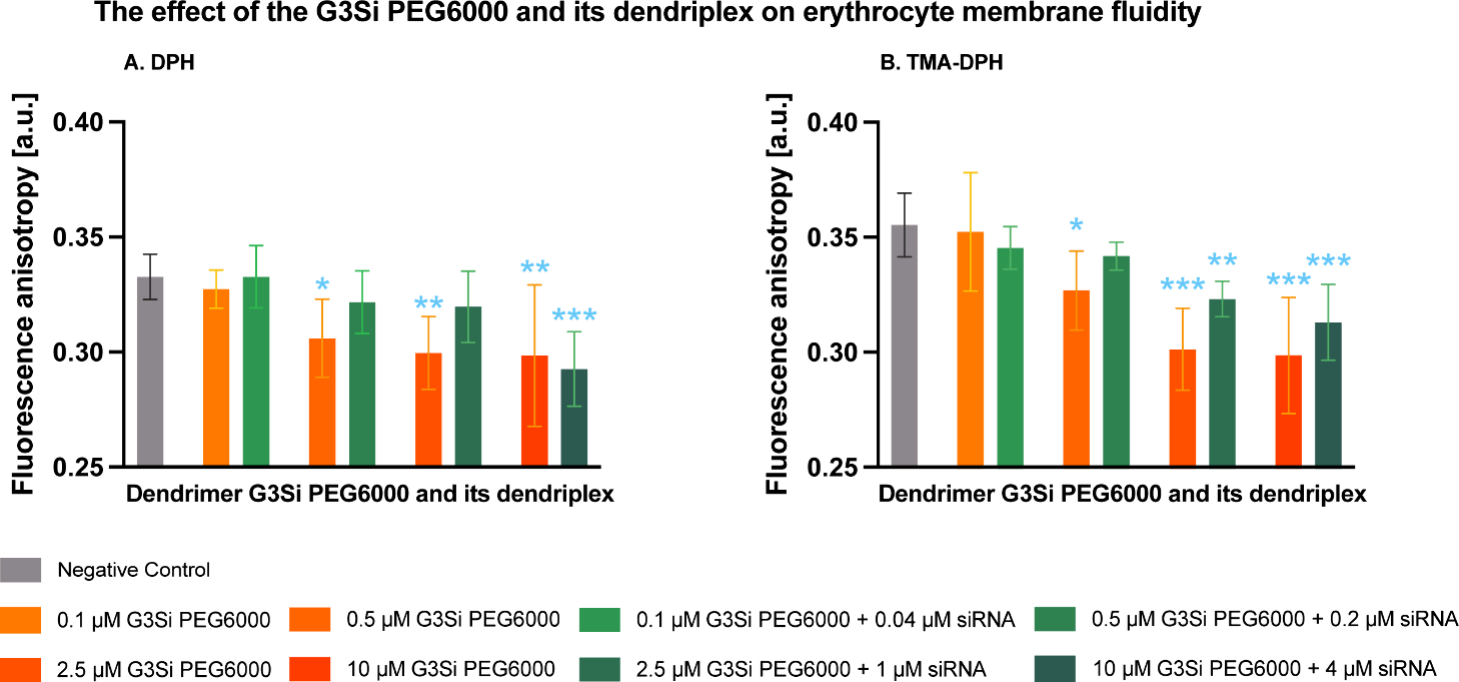
Fluorescence alterations of DPH (A) and TMA-DPH (B) probes indicate changes in membrane fluidity upon interaction with dendrimer G3Si PEG6000 and its dendriplex. A blue asterisk marks significant differences relative to the negative control.

### PBMC Cytotoxicity

Peripheral blood mononuclear cells (PBMCs) comprise lymphocytes (T cells, B cells, and NK cells), monocytes, and dendritic cells.^66^ PBMC cell viability was evaluated using the AlamarBlue assay, which measures the metabolic activity of PBMCs upon exposure to test compounds (Figure 6). This assay relies on the reduction of resazurin, a fully oxidized, blue, and nonfluorescent dye, into resorufin, a pink and fluorescent compound, through cellular redox reactions. Mitochondrial oxidoreductases mediate the reduction process, reflecting mitochondrial function and providing insights into cellular viability and proliferation.^67,68^ The assay results demonstrate no statistically significant cytotoxic effects following 24 and 48 h incubation with either the dendrimer or its dendriplex at any tested concentration when compared to the negative control. At the highest tested dendriplex concentration (10 μM), a statistically significant difference was observed between the 24 and 48 h time points, indicating a time-dependent normalization of cell viability. This temporal effect was not observed for the noncomplexed dendrimer, and a statistically significant difference between the dendrimer and dendriplex at the highest concentration suggests that complexation with siRNA may modulate the biological response. Notably, the only statistically significant difference relative to the negative control was an increase in PBMC viability, indicative of a proliferative response, following 48 h incubation with the lowest concentration of the noncomplexed dendrimer. Multiple nanoparticles have the capacity to induce the proliferation or death of PBMCs. These dual effects arise from complex interactions between the nanoparticles and cellular pathways.^69^ The minimal effect on PBMC viability is very beneficial, as it suggests the preservation of physiological immune functions without disrupting cellular processes. This property positions dendrimer G3Si PEG6000 as a promising candidate for biomedical applications, as it does not compromise the immune system by inducing cytotoxic effects, thereby preserving immunological homeostasis. While the absence of cytotoxicity toward PBMCs is an encouraging factor, comprehensive characterization of the immunological footprint of this nanosystem under physiologically relevant conditions is crucial for predicting systemic tolerance.

**Figure 6:**
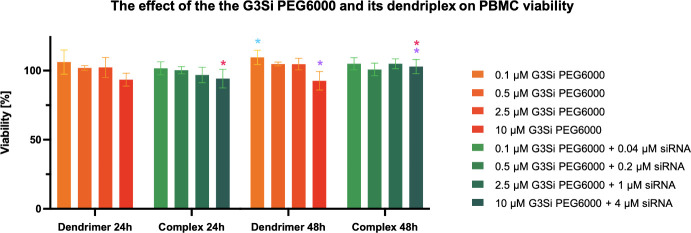
Viability of PBMCs treated with dendrimer G3Si PEG6000 and its dendriplex following 24 and 48 h incubation. A red asterisk denotes statistically significant effects attributed to incubation time, a violet asterisk indicates significant differences due to dendrimer-siRNA complexation, and a blue asterisk marks significant differences relative to the untreated cells (100%).

### PBMC Cytokine and Chemokine Expression Pattern

Nanoparticles can be recognized by antibodies and cellular receptors, thereby eliciting an immune response, inducing the release of cytokines and chemokines that regulate inflammation, drive immune cell recruitment, and influence cellular differentiation and activation. Biomolecular coronas can promote nanoparticle recognition by activating pattern recognition receptors (PRRs). PRRs may inadvertently cross-react with misfolded proteins or surface modifications that structurally mimic pathogen-associated molecular patterns (PAMPs) and damage-associated molecular patterns (DAMPs), resulting in activation of the immune response.^70^ While the biomolecular corona can induce both pro-inflammatory and anti-inflammatory signaling pathways, it can redefine a nanoparticle’s biological identity by masking synthetic surface features of possibly mitigating recognition by phagocytic cells and reducing the clearance rate from the bloodstream.^71–73^ Recent work reinforces that the formation of a plasma protein corona can markedly suppress nanoparticle-induced hemolysis and has spurred “corona-engineering” approaches by preloading nanoparticles with dysopsonins-rich layers such as clusterin or ApoA-I to stabilize cationic carriers and improve hemocompatibility.^74^ In light of the context-dependent cytokine modulation, we implemented a dual-environment experimental framework in both plasma-rich and plasma-poor environments. Evaluating both conditions in parallel, we ensure that the impact of adsorbed plasma proteins on the dendrimer surface identity and subsequent immune signaling is fully captured. Moreover, to more accurately recapitulate in vivo immunobiology, we extended our analysis beyond isolated cell types to include mixed cultures. This heterogeneous cellular milieu preserves critical paracrine interactions and cell–cell cross-talk, thereby revealing emergent cytokine networks and regulatory feedback loops that are inherently undetectable in single-cell assays. Collectively, this holistic strategy affords a deeper understanding of how the plasma milieu and native cellular complexity converge to dictate dendrimer-mediated immunomodulation.^75,76^ The selection of cytokines and chemokines was based on the interrelated roles, allowing observation of the influence that the studied dendrimer has on the inflammatory (IL-8, IL-12p40, TNFα), anti-inflammatory (IL-10), and pleiotropic (IFNα2, IL-6) immune signaling molecules (Figure 7).

**Figure 7:**
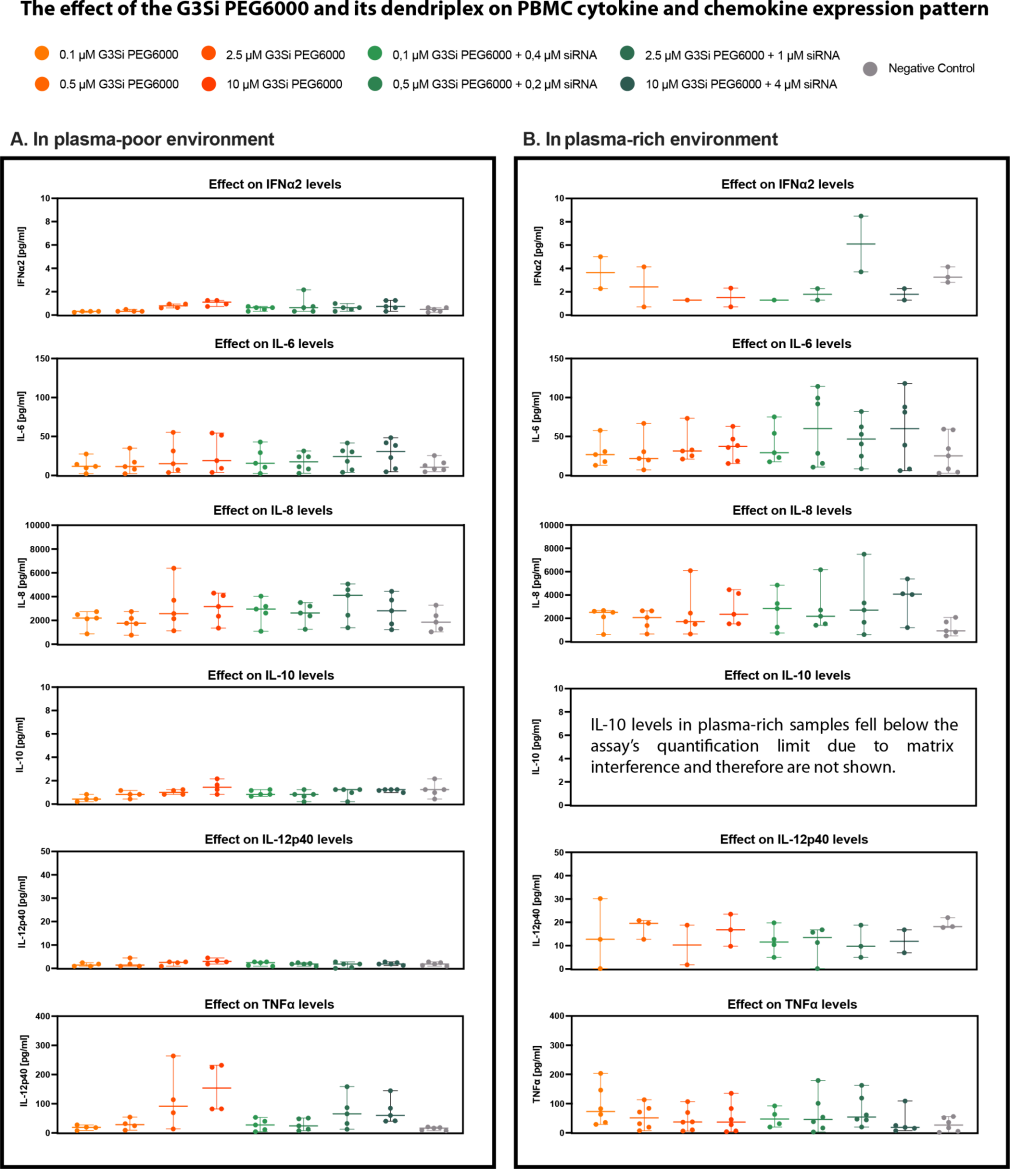
Effects of the G3Si PEG6000 dendrimer and its dendriplexes on cytokine and chemokine expression patterns. Panel A presents the effects of the nanosystem in the plasma-poor environment, whereas panel B illustrates the corresponding responses in the plasma-rich environment.

Our results suggest that introducing the nanoparticles into a plasma-rich environment led to a more heterogeneous and thus variable immune cell response, reflected by the cytokine and chemokine expression profiles. This variability of dendrimer G3Si PEG6000 is the most pronounced on IFNα2, IL-6, and IL-12p40 expression in plasma-rich environments, likely reflecting how the complex, dynamic, and heterogeneous protein corona modulates immune cell activation. In plasma-rich samples, IL-10 concentrations consistently fell below the lowest quantifiable concentration for the MAGPIX Luminex assay and were therefore excluded. The high protein content of plasma amplifies background noise, leading to substantial matrix interference that disproportionately diminishes the assay sensitivity for low-abundance analytes such as IL-10. In plasma-poor environments, the biomolecular corona is less complex, leading to a more consistent cellular activation pattern, if any. Our results indicate that under plasma-poor and plasma-rich environments, the G3Si PEG6000 dendrimer and its dendriplex induce IL-6 expression by PBMCs. In the plasma-poor environment, the highest tested dendrimer concentration mediated the release of IL-6 with a median reaching 19 pg/mL, while incubation with the highest tested dendriplex concentration resulted in a median equal to 30.76 pg/mL, representing accordingly a 1.80-fold and 2.9-fold increase compared to the negative control (10.54 pg/mL). In the plasma-rich environment, the median of IL-6 expression level reached 37.07 pg/mL for cells incubated with the highest tested dendrimer concentration and 60.06 pg/mL for samples incubated with the highest tested dendriplex concentration, representing accordingly a 0.6-fold and 2.4-fold increase compared to the negative control (25.17 pg/mL). Although these trends suggest an upregulation of IL-6 expression with higher concentrations of dendriplex, the differences did not achieve statistical significance. These observations, together with findings from several other studies, reinforce the concept that the siRNA cargo is not merely a passive therapeutic payload but plays a role in defining nanoparticle properties and modulating the resultant immune response.^77^ Moreover, our results suggest that the expression of IL-8 is modulated by both the dendrimer and its dendriplex in a manner that is dependent on the plasma environment. Under plasma-poor conditions, incubation of PBMCs with the highest concentration of the dendrimer G3Si PEG6000 resulted in the median of IL-8 expression level reaching 2855.59 pg/mL, corresponding to a 5.3-fold increase relative to the negative control, while the dendriplex further elevated the IL-8 median level to 3688.9 pg/mL, corresponding to a 6.9-fold increase compared to the negative control value. Conversely, in the plasma-rich environment, the dynamics shift occurs: the highest concentration of the studied dendrimer affected an IL-8 expression of median equal to 3171.46 pg/mL, representing a 1.7-fold increase over the control, while the incubation with the dendriplex led to a slightly lower level of 2814.09 pg/mL, representing a 1.5-fold increase relative to the negative control. In parallel to the trends observed for IL-8, the results suggest concentration-dependent effects of the tested nanosystem on TNFα expression in the plasma-poor environment, with the highest tested dendrimer concentration median reaching a 9.3-fold increase compared to the negative control value. In plasma-rich environments, however, the formation of a biomolecular corona masks the features of synthetic identity bioactive sites of the studied dendrimer, thereby reducing the dendrimer’s capacity to modulate TNFα level compared to the effects of the studied nanosystem in plasma-poor environments. This illustrates the critical role of the biomolecular corona in determining nanoparticle bioactivity and highlights the importance of testing nanoparticle effects under conditions that closely mimic the in vivo environment.

Quantitative assessment of cytokine and chemokine secretion profiles serves as a critical tool for evaluating the immunological safety of novel nanomaterials. Elevated levels of these mediators can act as early biomarkers of dysregulated immune activation, including the onset of cytokine storms, and may predict adverse outcomes such as chronic inflammation, autoimmune activation, or irreversible tissue injury. While no statistically significant elevations in cytokine or chemokine levels were observed when compared to negative controls, subtle yet reproducible shifts in expression patterns were detected. These findings suggest that the G3Si PEG6000 dendrimer and its dendriplex formulation may exert a mild immunostimulatory effect, indicating that they are not completely immunologically inert. Within this framework, PEG on G3Si PEG6000 may exert mixed effects. Under plasma-rich conditions, steric shielding by the biocompatible polymer can significantly reduce recognition and clearance of nanoparticles by the RES, which blunts some pro-inflammatory readouts.^78^ Conversely, PEG can also participate in complement-linked responses via anti-PEG antibodies, whose prevalence appears boosted in some populations after mRNA-LNP vaccination, potentially amplifying cytokine release in a donor-dependent manner.^79^ Nonetheless, owing to interdonor variation in anti-PEG antibodies and complex complement triggers, our data cannot predict the intensity of any anti-PEG response in vivo. The minimal pro-inflammatory signature observed here is a desirable characteristic for translational nanomedicine applications, as it supports the potential for prolonged systemic circulation and reduced risk of off-target immunotoxicity in vivo. Importantly, these findings lay a strong foundation for subsequent in vivo evaluations, where a subdued innate immune response is often a prerequisite for clinical feasibility.

### Hematological Blood Parameters

Building on the analyses conducted under simplified conditions, we expanded our investigation to evaluate the hemocompatibility of the G3Si PEG6000 dendrimer and its dendriplex in the complex environment of whole human blood. To achieve this, we utilized an advanced automatic hematology analyzer to study the effects of G3Si PEG6000 and its dendriplex on a comprehensive range of hematological parameters associated with RBCs, WBCs, and platelets (PLTs) over a 3 h incubation period.

The RBC parameters assessed—including RBC count (RBCC), hemoglobin (HGB), hematocrit (HCT), microcytic anemia factor (MAF), mean corpuscular volume (MCV), mean corpuscular hemoglobin concentration (MCHC), and red cell distribution width (RDW)—are critical indicators of erythrocyte health and function. In our study, the observation that these parameters remain within normal ranges, with minimal deviation from control samples, suggests that the introduction of G3Si PEG6000 dendrimers and their dendriplexes does not adversely affect red blood cells (Figure 8). Specifically, the unaltered RBC count and HGB, HCT, and MAF levels suggest the absence of eryptosis and agree with the results obtained in the hemotoxicity experiment in the presence of plasma. Stable MCV, MCHC, and RDW values further imply that the dendrimer exposure does not cause pronounced morphological alterations or cellular heterogeneity in RBC.

**Figure 8:**
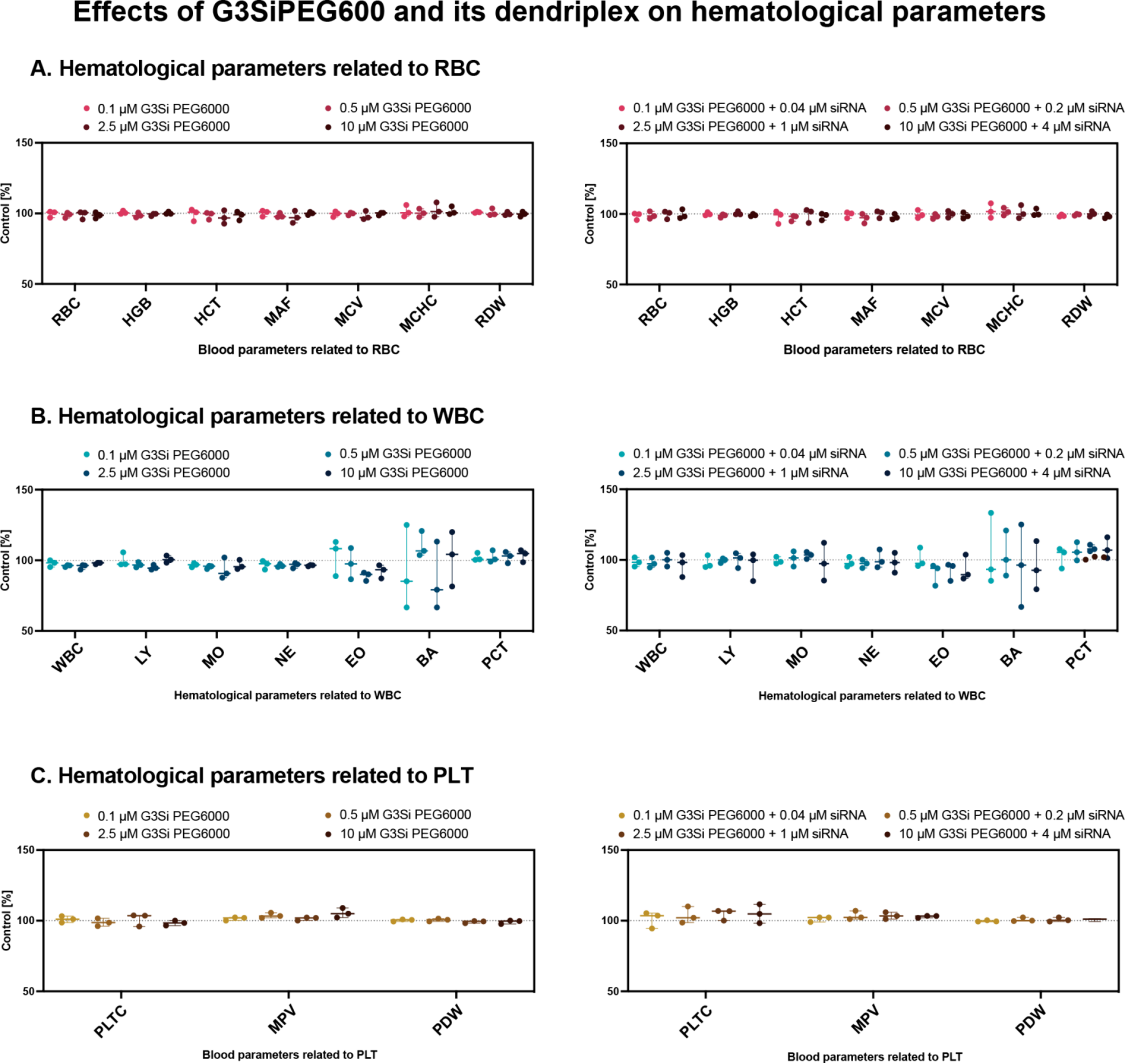
Effects of the G3Si PEG6000 dendrimer and its dendriplexes on various hematological parameters, expressed as a percentage relative to the negative control. Each panel pair in the figure represents parameters associated with RBC (A), WBC (B), and PLT (C). Individual data points correspond to measurements from single patients, and the horizontal line within each panel indicates the median value of the respective parameter.

The evaluation of WBC parameters—including total WBC count (WBCC), neutrophils (NE), lymphocytes (LY), monocytes (MO), eosinophils (EO), basophils (BA), and procalcitonin (PCT) levels—revealed that most values remained within normal ranges. However, the observed variability in eosinophil and basophil counts may be attributed to analytical imprecision inherent in automated hematology analyzers. Basophils, being the least abundant WBC, present a challenge for precise quantification. Several studies have indicated that automated analyzers often exhibit high analytical imprecision and poor specificity in basophil counting and, to a lesser extent, for eosinophils.^80,81^ Therefore, the scattered results observed for basophil and eosinophil counts in this study likely reflect the known limitations in the precision of automated hematology analyzers rather than an actual effect of dendrimer or dendriplex exposure. These results are in line with the results obtained in PBMC toxicity. Additionally, PCT levels remained within normal ranges, indicating that the nanovector exposure did not elicit a systemic inflammatory response, mimicking systemic bacterial infection.

The evaluation of PLT parameters—including PLT count (PLTC), mean platelet volume (MPV), and platelet distribution width (PDW)—revealed that all values remained within normal ranges, exhibiting minimal deviation from control samples. This consistency suggests that introducing G3Si PEG6000 dendrimers and their dendriplexes into whole blood does not adversely affect PLT function or morphology. Nanovectors may exert diverse effects on platelet behavior. Nanoparticles with negative surface charges, smaller sizes, and lower molecular weights have been shown to inhibit PLT aggregation. Conversely, cationic dendrimers are known to stimulate platelet aggregation due to the electrostatic interaction between their positively charged surfaces and the negatively charged platelet membrane. The observed stability of platelet parameters in the presence of positively charged G3Si PEG6000 dendrimers and its dendriplex may be attributed to the nanovector’s PEGylation. It is well established in the scientific literature that surface modification reduces interactions with platelets and other blood components while enhancing biocompatibility, thereby minimizing the risk of adverse effects.^82^

### Coagulation Blood Parameters

Advanced optical automatic hemostasis analyzer was utilized to evaluate the effect of the G3Si PEG6000 dendrimer and its dendriplex on coagulation parameters—including thrombin time (TT), quantitative fibrinogen assay (QFA), prothrombin time (PT), and activated partial thromboplastin time (aPTT)—demonstrated that TT, QFA, and PT values remained within normal ranges, exhibiting minimal deviation from control samples (Figure 9). However, a notable finding was the concentration-dependent prolongation of aPTT observed with the addition of both the G3Si PEG6000 dendrimer and its dendriplex. The isolated prolongation of aPTT, with physiologically normal values of TT, QFA, and PT, suggests the impact of the tested nanosystem on the intrinsic pathway of the coagulation cascade. The control aPTT median was 35.2 s, which increased to 42.9 s in samples containing 2.5 μM dendrimer and further to 74.4 s at a dendrimer concentration of 10 μM. In comparison, the aPTT prolongation was less pronounced in samples containing the dendriplex. The median aPTT for samples with 2.5 μM G3Si PEG6000 dendrimer and 1 μM siRNA rose to 37.5 s, while those with 10 μM G3Si PEG6000 dendrimer and 2.5 μM siRNA exhibited a median aPTT of 47.6 s. Prolongation of the aPTT by the tested nanovector may result from its interaction with coagulation factors specific to the intrinsic pathway. This interaction likely involves binding these factors to the dendrimer surface, leading to their depletion or functional inhibition. Multiple studies have demonstrated that nanoparticles can affect the coagulation pathways in various ways. Similar aPTT-biased anticoagulant effects are reported for polydopamine nanoparticles and ZIF-8 MOFs, reinforcing that nanosurface interactions can sequester intrinsic-pathway factors without broad extrinsic disturbance.^83,84^ Notably, cationic amine-terminated PAMAM dendrimers were found to induce disseminated intravascular coagulation (DIC)-like effects in vivo at high doses.^85^ The ability of nanovectors, such as PAMAM dendrimers, to interact with coagulation pathways underscores the critical importance of comprehensive safety testing in nanomedicine development. The mechanism of DIC itself is highly complex, involving coagulation factors, leukocytes, endothelial cells, and platelets, which collectively drive the pathological cascade. While the tested dendrimer G3Si PEG6000 and its dendriplex demonstrated no substantial effects on these critical components, suggesting a low likelihood of DIC development, future study designs and analyses must account for the likelihood of resulting in interruption of the intrinsic coagulation pathway to ensure their safety for effective application.

**Figure 9:**
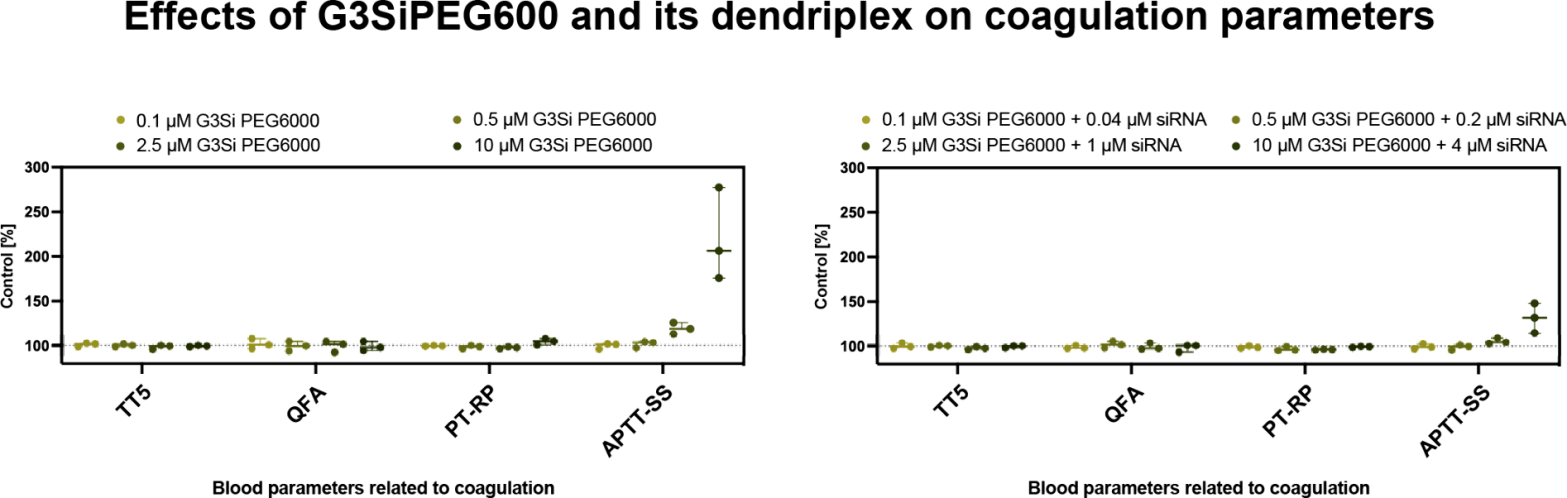
Effects of the G3Si PEG6000 dendrimer and its dendriplex on coagulation parameters, expressed as a percentage relative to the negative control. The panel on the left displays the effects of the dendrimer, while the panel on the right shows the effects of the dendriplex. Individual data points correspond to measurements from single patients, while the horizontal line within each panel indicates the median value of the respective parameter.

## Conclusions

Given the increasing number of nanotechnology-based products in the development, evaluating the effects of nanoparticles on blood components is essential, as these interactions can significantly influence both the fate and efficacy of the nanovectors and constitute risks to hematological balance. Our comprehensive in vitro investigation of dendrimer G3Si PEG6000 and its dendriplex provides critical insights into the interactions between the nanovector and blood elements, highlighting the importance of biomolecular corona formation. The study conclusively demonstrates that dendrimer complexation with siRNA and biomolecular corona formation effectively mitigates direct hemolytic damage. Increasing concentrations of dendrimer G3Si PEG6000 and its dendriplex induce a modest, concentration-dependent increase in blood viscosity, most notably at lower shear rates. While the viscosity rise is comparable between the two formulations, the observed changes remain below thresholds typically associated with significant cardiovascular risks. Fluorescence spectroscopy analysis revealed that exposure to G3Si PEG6000 dendrimer and its dendriplex increases erythrocyte membrane fluidity, as evidenced by a decrease in fluorescence anisotropy. The uncomplexed dendrimer exerts a pronounced effect on membrane dynamics, whereas siRNA complexation attenuates this effect, thereby reducing dendrimers impacts membrane integrity. These findings align with hemotoxicity studies and suggest the hemorheology changes likely arise from enhanced cell-to-cell adhesion. In vitro cytotoxicity assays confirm that both the dendrimer and its dendriplex exhibit minimal effects on PBMC viability over 24 and 48 h incubation periods relative to the negative control, with only transient, nonsignificant variations observed at the highest concentrations. Modest immunomodulatory responses were observed, particularly reflected in cytokine expression profiles such as IL-6, IL-8, and TNFα; however, these remained within ranges unlikely to trigger severe systemic immune reactions. Notably, the magnitude and nature of these responses were strongly influenced by the presence of plasma and the siRNA cargo, highlighting the critical role of both the biological milieu and therapeutic payload in shaping the immunological behavior of the nanocarrier—an essential consideration for the design and clinical translation of any nanosystem. Advanced hematological analyses indicate that the nanovectors do not adversely alter RBC, WBC, or PLT parameters. Notably, most coagulation parameters remained within physiological limits, except for a nonsignificant, concentration-dependent prolongation of the aPTT, which suggests a mild impact on the intrinsic coagulation cascade. This effect was less pronounced in samples incubated with the dendriplex, likely due to siRNA complexation mitigating direct interactions with coagulation factors. A summary of the key findings is presented in Table 1. Ultimately, our data also highlight specific areas of concern that warrant further investigation in future studies, ensuring comprehensive safety profiling is necessary for effective clinical translation. Collectively, these results underscore the translational potential of this nanosystem for therapeutic applications and emphasize the importance of therapeutic cargo and biomolecular corona formation in assessing nanocarriers’ safety and efficacy.

**Table 1:**
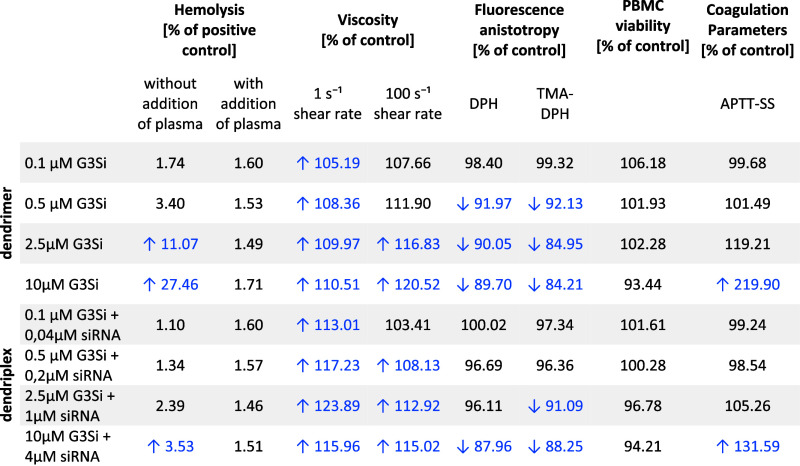
Summary of Selected Hemocompatibility Parameter Readouts for the G3Si PEG6000 Dendrimer and Its siRNA Dendriplexes, Chosen from a Broader Panel of Parameters Evaluated in This Manuscript^a^

a Hemolysis and PBMC viability shown here were measured after 24 h of incubation; hemolysis is expressed as % of the positive control (100% RBC lysis), whereas viscosity (1 and 100 s^–1^), fluorescence anisotropy (DPH, TMA-DPH), PBMC viability, and APTT-SS are expressed as % of the negative control. Blue arrows (↑/↓) indicate the direction of statistically significant changes relative to the corresponding control.

## Abbreviations

APOE4: apolipoprotein E 4
aPTT: activated partial thromboplastin time
BA: basophils
BBB: blood–brain barrier
CNS: central nervous system
DAMPs: damage-associated molecular patterns
DIC: disseminated intravascular coagulation
DPH: 1,6-diphenyl- 1,3,5-hexatriene
EMA: European Medicines Agency
EO: eosinophils
FBS: fetal bovine serum
FDA: Food and Drug Administration
HCT: hematocrit
HGB: hemoglobin
LY: lymphocytes
MAF: microcytic anemia factor
MCHC: mean corpuscular hemoglobin concentration
MCV: mean corpuscular volume
MO: monocytes
MPS: mononuclear phagocytic system
MPV: mean platelet volume
NE: neutrophils
PAMPs: pathogen-associated molecular patterns
PBMC: peripheral blood mononuclear cell
PBS: phosphate-buffered saline
PCT: procalcitonin
PDW: platelet distribution width
PEG: polyethylene glycol
PLTC: PLT count
PLT: platelets
PRRs: pattern recognition receptors
PT: prothrombin time
QFA: quantitative fibrinogen assay
RBCC: RBC count
RBC: red blood cells
RDW: red cell distribution width
RES: reticuloendothelial system
TMA-DPH: N,N,N-trimethyl-4-(6-phenyl-1,3,5-hexa-triene-1-yl)
TT: thrombin time
WBCC: total WBC count
WBC: white blood cell
